# User survey finds rapid evidence reviews increased uptake of evidence by Veterans Health Administration leadership to inform fast-paced health-system decision-making

**DOI:** 10.1186/s13643-016-0306-5

**Published:** 2016-08-05

**Authors:** Kim Peterson, Nicole Floyd, Lauren Ferguson, Vivian Christensen, Mark Helfand

**Affiliations:** 1 VA Portland Health Care System, Veterans Affairs Evidence-Based Synthesis Program, 3710 SW U.S. Veterans Hospital Road, Portland, OR 97239 USA; 2 Oregon Health and Science University, 3181 S.W. Sam Jackson Park Rd., Portland, OR 97239 USA

**Keywords:** Rapid review, Evidence synthesis, Decision-making, Implementation, Program impact

## Abstract

**Background:**

To provide evidence synthesis for faster-paced healthcare decision-making, rapid reviews have emerged as a streamlined alternative to standard systematic reviews. In 2012, the Veterans Affairs Evidence-based Synthesis Program (VA ESP) added rapid reviews to support Veterans Health Administration (VHA) operational partners’ more urgent decision-making needs. VHA operational partners play a substantial role in dissemination of ESP rapid reviews through a variety of routes, including posting on the VA ESP’s public website (http://www.hsrd.research.va.gov/publications/esp/). As demand for rapid reviews rises, much progress has been made in characterizing methods and practices. However, evidence synthesis organizations still seek to better understand how and when rapid reviews are being used.

**Methods:**

The VA ESP administered an online survey to rapid review operational partners. The survey assessed the nature of decision-making needs, overall perception of review content, resulting actions, and implementation timeframe. We use descriptive statistics and narrative methods to summarize findings.

**Results:**

Between October 2011 and April 2015, we completed 12 rapid reviews for 35 operational partners. Operational partners were primarily non-academic subject matter experts with VA operations’ decision-making authority. The most common topic categories reviewed were policy or system (50 %) or process of care (42 %) initiatives. Median report completion time was 14.5 weeks. Survey response rate was 46 %, with at least one operational partner responding for 92 % of reports. Reviews served multiple purposes including policy directive or regulation (72 %), supporting program development and evaluation (55 %), identifying future research needs (45 %), and determining implementation strategy (45 %). Overall, operational partners’ perception of report content was positive. A majority of rapid reviews were used immediately and informed actions ranking high on the Institute of Medicine’s Degrees of Impact framework: 45.4 % effected change, 18.2 % inspired action, 18.2 % informed the field, 9.1 % received recognition, and 9.1 % spread a message.

**Conclusions:**

VA ESP rapid reviews have increased the VHA’s uptake of evidence to inform time-sensitive system-level decision-making. Key areas of interest for future evaluation include assessing user perception of our streamlined methods and the quality of our efforts to inform users of these methods, as well as comparing the usability and impact of our rapid and standard systematic reviews.

**Electronic supplementary material:**

The online version of this article (doi:10.1186/s13643-016-0306-5) contains supplementary material, which is available to authorized users.

## Background

To meet time-sensitive demands for quality evidence, rapid reviews have emerged as a streamlined alternative to standard systematic reviews [1–7]. Rapid reviews are used by a variety of health organizations such as Kaiser Permanente, Blue Cross Blue Shield Association, and University of Pennsylvania Health System, and the demand for them only continues to increase [1, 3, 4, 8]. Rapid review products use various approaches to abbreviate the systematic review process. Overall, the two main sources of variation are timeframe (days to months) and extent of synthesis (none to both qualitative and quantitative). Hartling et al. [4] grouped rapid review products into the following four categories based on the extent of synthesis: (1) “inventories” provide a listing of the available evidence, within 3 days to 6 months; (2) “rapid responses” present the best available evidence with no formal synthesis, within 5 days to 3 months, and often rely on secondary sources; (3) “rapid reviews” synthesize the quality of and findings from the evidence, generally within 2 to 4 months; and (4) “automated approaches” generate meta-analyses in response to user-defined queries.

Other common methods of streamlining the systematic review process include limiting literature searches, extent of data abstraction and quality appraisal, and the use of dual independent reviewing [9]. Although rapid reviews carry the promise of increasing the uptake of evidence in healthcare decision-making where the alternative is no evidence at all [1, 2, 5, 10], uncertainty remains about their potential trade-offs. Concern has been raised that streamlining may compromise the quality of the work and increase the risk of missing evidence or errors in the synthesis, ultimately decreasing utility to end users [3, 4].

Interviews with rapid review producers identified the “continuous intimate relationship with a specific end user” and the nature of the decision as key drivers of rapid review approaches [4]. Rapid reviews also usually require reaching a consensus quickly, which promotes involving stakeholders from different backgrounds early in the process and invites closer attention throughout the revisions [3]. While much progress has been made over the past several years in characterizing rapid review methods and current practices [2, 4–6], less is known about the users of rapid reviews, their knowledge and acceptance of the streamlined methods used to produce rapid reviews, and the impact rapid reviews are having on health system decision-making [11].

To gain insight into users’ acceptance of methods used to streamline the systematic review process, an Agency for Healthcare Research and Quality (AHRQ) Evidence-based Practice Center (EPC) Method Workgroup conducted qualitative interviews of eight frequent and known users (“Key Informants”) of EPC standard systematic reviews [12]. Key Informants evaluated three sample rapid products on venous thromboembolism and gave their impressions on streamlining approaches and how they might use such products. In exchange for shorter review timelines, the majority of Key Informants were willing to have shortcuts made in the literature search (such as limiting databases, journals, years) and in the abstract and full-text review process (such as using a single reviewer) rather than independent review by two reviewers. However, Key Informants also noted that as potential users may not be aware of the potential ramifications of streamlining standard systematic review methods, rapid review producers have a responsibility to help educate users about the process. Finally, Key Informants identified credibility of the review producer and strength of evidence assessments as critical components of a rapid review. Compared to the eight Key Informants interviewed, however, less-frequent users of reviews or varied audiences may have different perspectives.

Previous evaluations of the impact of rapid reviews have largely focused on health technology topics used to inform Canadian provincial healthcare system coverage and acquisition decisions [13–15]. These evaluations found that rapid health technology assessments (HTAs) have consistently influenced policy decisions, including use as reference material and incorporation of the assessment’s recommendations and conclusions [13, 14]. Similarly, the University of Pennsylvania Health System’s Center for Evidence-based Practice reported that the majority of their rapid technology reviews actually informed users’ final clinical practice, policy, purchasing, and formulary decisions [8]. Additionally, in Quebec, the budget impact of rapid HTAs developed on-site in collaboration with end users was estimated at approximately $3 million in savings per year [15]. Although these rapid HTAs were seen as useful, some authors acknowledged that they were typically considered only as interim products that should be followed up with full assessments. This is because the short timeframes increased the chance of providing inappropriate advice and typically restricted the scopes to only addressing questions of efficacy or effectiveness [14].

These studies provide preliminary information on the use and influence of rapid HTAs in a few specific settings. As healthcare decision-makers are increasingly demanding accelerated forms of evidence synthesis, rapid reviews are meeting an important need within health systems. It is important, however, to better understand when and in what capacity rapid reviews are used, as well as the mechanisms that help or hinder their implementation from the user’s perspective for a broader range of topics and settings [11].

The Veterans Affairs’ Evidence-based Synthesis Program (VA ESP) was established in 2007 to provide the Veterans Health Administration (VHA) with timely and accurate evidence synthesis on important topics to meet their healthcare decision-making needs and to improve Veterans’ health and healthcare [16]. The VA Quality Enhancement Research Initiative (QUERI) provides funding for four ESP Centers, and each Center has an active university affiliation with close ties to the AHRQ Evidence-based Practice Center Program. The Centers are located at the Durham and West Los Angeles VA Medical Centers, the Minneapolis VA Health Care System, and the VA Portland Health Care System. The ESP Coordinating Center (ESP CC), also located in Portland, oversees national ESP program operations, program development and evaluation, and dissemination efforts. Each Center is led by a VA clinician investigator and staffed with 2–3 FTE research assistant/associates. The Centers rely heavily on fellows and residents to round out review teams, and each produces 3 standard systematic reviews annually.

In 2012, in response to VHA operational partner feedback, the VA ESP added rapid reviews to support the VHA’s more urgent decision-making needs [17]. The ESP CC increased capacity to provide this product and added a dedicated research staff with extensive systematic review expertise. The rapid review team is led by a VA clinician researcher (.10 FTE) and consists of 1.6 FTE research associates, .50 FTE librarian, and .50 FTE research assistant and utilizes the support of existing ESP CC infrastructure including a full-time Associate Director charged with program management and an editorial coordinator. The ESP CC conducts 3 to 5 rapid reviews each fiscal year. Consequently, rapid reviews are reserved for topics which (1) are identified as top priority by senior management, (2) would potentially have important consequences if delayed, and (3) have a mechanism in place that will allow for rapid implementation of findings. VA ESP rapid review products are completed within 4 months, include primarily qualitative syntheses and conclusions that rely on internal validity and strength of evidence assessments, and are subjected to external peer review, which best resemble the “rapid review” type of products from the taxonomy described above [4]. Our primary means of gaining efficiency is by tailoring the scope to focus on parameters that would drive the operational partners’ decision-making (for example, health outcomes vs intermediate outcomes). Depending on the volume of evidence and time allowed, other steps may be taken to abbreviate the review process, including substituting the second reviewer verification of study selection, data abstraction, quality assessment, and strength of evidence ratings for dual independent review. The VA ESP rapid reviews are led by experienced systematic reviewers who draw on core systematic review values of focusing on the highest-quality evidence, minimizing bias, and maximizing transparency to make decisions about how to abbreviate processes. ESP rapid reviews have primarily addressed process of care, access topics, and systems policy initiative needs.

Operational partners play a substantial role in dissemination of ESP rapid reviews through a variety of routes. All rapid reviews are posted on the VA ESP’s public program website (http://www.hsrd.research.va.gov/publications/esp/) and indexed in PubMed and may be submitted for publication in peer-reviewed journals where appropriate. The ESP CC consults with operational partners to develop a tailored plan for each report, identifying appropriate strategies that are topic-specific and targeted to optimize uptake by the health system. Dissemination efforts may include (1) VA Cyberseminars (i.e., national, online, free, video-archived presentations of report findings), which are augmented by policy and clinical work in order to make the presentations relevant and applicable to clinicians, administrators, and researchers and (2) presentation of findings at leadership briefings, program/committee meetings, or conferences. Operational partners also frequently recommend dissemination strategies and targets for “Management eBriefs,” an electronic publication to provide VHA management with a concise summary of report findings, including implications for VHA policy or practice.

In early 2015, the VA ESP initiated a quality improvement effort aimed at understanding the utility of the evidence products and their impact on decision-making in the VHA. The project involves surveying operational partners—high-level VHA leadership that request and use the evidence products—regarding (1) the nature of their decision-making needs, (2) actions resulting from the report’s findings, (3) implementation timeframe, and (4) overall perception of report content. These objectives were inspired by the VHA’s and QUERI’s goals of rapidly translating research findings and evidence-based treatments into clinical practice, increasing the impact of VA research findings through evaluation, and promoting the VHA as a learning healthcare organization through innovative implementation science [18]. In this article, we report on the retrospective survey results for 11 (out of 12) rapid reviews completed between 2011 and 2015. Our survey findings extend knowledge on users’ perspectives of how and when they use rapid reviews to different types of users, settings, and report topics than have been previously evaluated.

## Methods

The VA ESP CC drafted the initial survey instrument based on the QUERI Strategic Plan, the VHA Strategic Plan (“Blueprint for Excellence”), and their linkage to the goals of the ESP. The ESP CC refined the survey based on feedback from the Directors of the ESP Centers as well as VA research and implementation leadership. The final survey assessed the following: (1) nature of decision-making needs, (2) actions resulting from the report’s findings, (3) implementation timeframe, and (4) overall perception of report content. The survey comprised both open- and closed-ended questions, to encourage respondents to provide in-depth detail regarding the quality of the review’s content and actions taken as a result of the report findings (see Additional file 1). We administered the survey using Survey Monkey (SurveyMonkey Inc. Palo Alto, CA), an online, cloud-based survey creation and administration tool. The survey was reviewed and approved as quality improvement based on VHA policy [19].

Study participants were operational partners, defined as leaders of a VHA national program office or business line who are responsible for national clinical programs or policies in the deployment of VHA health services. We surveyed all 35 operational partners who requested all 12 VA ESP rapid reviews we produced from 2011 to 2015. We recruited operational partners via an email that included a link to the online survey. In the recruitment email, we notified operational partners that we would keep their identities confidential. We sent the surveys out in four groups between July and October of 2015. We gave operational partners 4 weeks to respond. We sent nonrespondents a reminder email at 14 days. We compared survey respondents and nonrespondents with respect to their organizational role: (1) Academic Researchers charged with leading system-wide health/quality improvement efforts (no VA operation decision-making authority), (2) non-academic Subject-Matter Experts (SME) with VA operation decision-making authority, including National Program Offices, Central Office, and Chief Consultants, or (3) non-academic Health System Managers with VA operation decision-making authority, such as VISN Directors or Chief Medical Officers.

We imported survey results into Microsoft Excel (Microsoft Corp, Redmond, WA) and used Stats Direct Version 2.8.0 (CamCode, UK) for analysis. We conducted statistical comparisons using *χ*^2^ and Fisher’s exact tests. Narrative methods were used to analyze open-ended responses. We organized the open-ended responses about actions resulting from the report based on the Institute of Medicine’s (IOM) Degrees of Impact—a scale intended to gauge impact made in health systems [20]. This scale provides metrics for assessing five levels of impact: (1) effecting change (e.g., revision of guidelines, legislation enacted), (2) inspiring action (e.g., legislation introduced, advocacy initiatives), (3) informing the field (e.g., subject of meeting or hearing), (4) receiving recognition (e.g., formal response by stakeholders), and (5) spreading the message (e.g., published article). For open-ended responses about how ESP reports compared with other evidence sources, we categorized them as (1) compares equally/similar, (2) prefers ESP for VA focus, (3) no opinion, and (4) other. Open-ended responses were initially coded by one reviewer and verified by one or two other reviewers. Disagreements were resolved by consensus. Close-ended responses were evaluated using descriptive statistics. We planned to explore heterogeneity in operational partners’ perception of content as potential sources of variability in report impact.

## Results

### Review characteristics

Between 2011 and 2015, we completed 12 rapid reviews for 35 operational partners (Table 1). Reviews had on average three operational partners (range, 1 to 9). The majority (94 %) of operational partners were non-academic SMEs and two (6 %) were non-academic Health System Managers. Overall, the majority of reviews examined policy or organizational/managerial system topics (50 %), defined as “a report primarily examining laws or regulations; the organization, financing, or delivery of care, including settings of care; or healthcare providers,” or process of care topics (42 %), defined as “a report primarily examining a clinical pathway or a clinical practice guideline that significantly involves elements of prevention, diagnosis, and/or treatment” [8]. Due to limited and heterogenous evidence, only two rapid reviews provided opportunities to perform original meta-analyses of small numbers of studies (repetitive transcranial magnetic stimulation for treatment-resistant depressions, updates on the prevalence of and interventions to reduce racial and ethnic disparities). We have increased our use of strength of evidence assessments over time, for an overall rate of 67 %. Median time to report completion was 14.5 weeks overall, increasing from 9 weeks in the first year to 20 weeks in the fourth year to more regularly incorporate strength of evidence assessment and peer review processes. Beyond posting reviews on our public website, additional dissemination activities varied across topics and time.

**Table 1:** Summary of review topic categories, methodology, timeframe, and dissemination by fiscal year (FY)

	Overall (*N* = 12)	FY12 (*N* = 2)	FY13 (*N* = 5)	FY14 (*N* = 4)	FY15 (*N* = 1)
Median report completion time (in weeks)	14.5	9	15	15	20
Report topic category					
Policy or organizational/managerial system^a^	6 (50 %)	0	4 (80 %)	2 (50 %)	0
Process of care^b^	5 (42 %)	2 (100 %)	1 (20 %)	1 (25 %)	1 (100 %)
Device	1 (8 %)	0	0	1 (25 %)	0
Methodology					
Performance of original meta-analyses	2 (17 %)	0 %	0 %	1 (25 %)	1 (100 %)
Performance of strength of evidence assessments	8 (67 %)	1 (50 %)	3 (60 %)	3 (75 %)	1 (100 %)
Dissemination					
Publically available on VA website	12 (100 %)	2 (100 %)	5 (100 %)	4 (100 %)	1 (100 %)
Management eBriefs	3 (25 %)	1 (50 %)	0 %	1 (25 %)	1 (100 %)
Cyberseminars	1 (8 %)	1 (50 %)	0 %	0 %	0 %
Peer-reviewed journal submission in process	3 (25 %)	0	1 (20 %)	1 (25 %)	1 (100 %)
Presentation of findings at leadership briefings, program/committee meetings, or conferences	5 (42 %)	1 (50 %)	3 (60 %)	0 %	1 (100 %)

^a^A report primarily examining laws or regulations; the organization, financing, or delivery of care, including settings of care; or healthcare providers [8]

^b^A report primarily examining a clinical pathway or a clinical practice guideline that significantly involves elements of prevention, diagnosis, and/or treatment [8]

### Survey findings

**Fig. 1:**
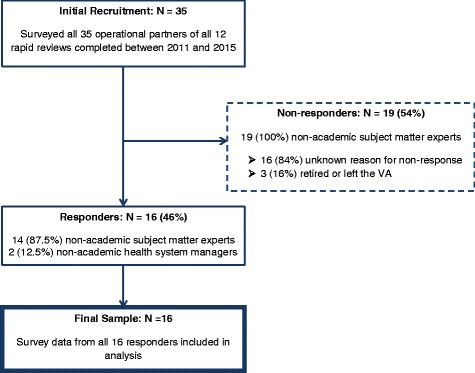
Survey respondents, non-respondents, and response rates

Survey response rate was 46 % (range, 0 to 100 % per review) (Fig. 1). Eleven out of 12 reviews had at least one operational partner respond (92 %). Data was completed and analyzed for all 16 respondents. Nineteen of 35 (54 %) operational partners did not respond to the survey (Fig. 1). The proportions of Health System Managers were similar between responders and nonresponders (12.5 vs 0 %; *P =* .20). Respondents were asked to select the variety of ways in which they were involved in the review process. Sixty-nine percent provided input on the scope of the review, 63 % gave feedback on the draft report, 63 % were briefed on the report’s findings, and 50 % had periodic contact throughout the review process.

Table 2 displays the types of reports, findings, and what the user perceived as their primary purpose. Respondents indicated that reviews served multiple purposes—an average of 2.25 purposes per report. The most common review purposes were the following: policy directive or regulation (72 %), supporting program development and evaluation (55 %), identifying future research needs (45 %), and determining implementation strategies (45 %).

**Table 2:** Report characteristics

Report title	Survey response rate	Operational partners’ description of report purpose	Timeline and final report date	General findings	Link to report
Role of the annual physical examination in the asymptomatic adult	0/1 (0 %)	No response obtained	6 weeks	Comprehensive routine physical examinations are not recommended for the asymptomatic adult.	http://www.hsrd.research.va.gov/publications/esp/physical.cfm
			Oct 2011		
Effect of geriatricians on outcomes of inpatient and outpatient Care	2/3 (67 %)	Determine implementation strategy; guideline or directive; support resource allocation decisions; clinical guidance	12 weeks	The impact of geriatrician involvement on patient function and healthcare utilization varies across the different models of care that include geriatricians in different roles.	http://www.hsrd.research.va.gov/publications/esp/geriatricians.cfm
			May 2012		
Effectiveness of intensive primary care programs	2/9 (22 %)	Clinical guidance; identify future research needs; support program development and evaluation activities	16 weeks	Inconsistent findings on whether these models of care reduced hospitalizations.	http://www.hsrd.research.va.gov/publications/esp/primary-care.cfm
			Nov 2012		
Developing a threshold for small VA hospitals	1/4 (25 %)	Guideline or directive; identify future research needs; determine implementation strategy	12 weeks	A relationship between hospital size and quality measures was either not found (for adverse events) or was inconsistent (for other measures).	http://www.hsrd.research.va.gov/publications/esp/hospital-size.cfm
			Feb 2013		
Effects of small hospital closure on patient outcomes	1/2 (50 %)	Resource allocation decisions	15 weeks	Low-strength evidence that hospital closures leading to increased distance and/or time to nearest hospital may increase mortality for time-sensitive conditions.	http://www.hsrd.research.va.gov/publications/esp/hospital-closure.cfm
			May 2013		
Relationship between time delay to colonoscopy and colorectal cancer outcomes	3/5 (60 %)	Guideline or directive; clinical guidance; determine implementation strategy	16 weeks	No evidence to support current policy requiring follow-up colonoscopy within 60 days of positive screening fecal occult blood tests.	http://www.hsrd.research.va.gov/publications/esp/fecaloccult.pdf
			May 2013		
Review of reviews on specialty care topics	1/3 (33 %)	Program development and evaluation activities	4 weeks	Provided inventory of main findings from systematic reviews on the topics of shared decision-making in palliative care, oncology, and nephrology; interventions that reduce hospitalizations/emergency room (ER) visits for heart failure and chronic obstructive pulmonary disease (COPD); and interdisciplinary specialty care platforms/teams/neighborhood approaches for reducing hospitalizations/ER visits.	http://www.hsrd.research.va.gov/publications/esp/specialty-care.cfm
			July 2013		
Effectiveness of mandatory computer trainings on ethical, workplace, and security topics	1/1 (100 %)	Performance measure; update existing review; determine implementation strategy; support program development and evaluation activities	14 weeks	No studies identified.	http://www.hsrd.research.va.gov/publications/esp/mandatory-training.cfm
			May 2014		
Primary care initial appointment wait times threshold	1/1 (100 %)	Guideline or directive	6 weeks	No clear support for broad use of any specific wait time standard for new patients in accessing their first primary care or mental health appointment. Offered potential options for selecting a wait time target.	http://www.hsrd.research.va.gov/publications/esp/wait-time.cfm
			July 2014		
Factors that optimize therapy with repetitive transcranial magnetic stimulation for treatment-resistant depressions	1/3 (33 %)	Clinical guidance	16 weeks	High-frequency rTMS applied to the left dorsolateral prefrontal cortex is the best-studied approach and it includes a FDA-cleared protocol that has been shown to improve quality of life.	http://www.hsrd.research.va.gov/publications/esp/rtms.cfm
			July 2014		
Quality of care provided by advanced practice nurses	1/2 (50 %)	Inform proposed regulation	24 weeks	Low-strength evidence suggesting no difference in health status, quality or life, mortality, or hospitalizations favoring either APRN or physician care in primary or urgent care settings.	http://www.hsrd.research.va.gov/publications/esp/ap-nurses.cfm
			Sept 2014		
Updates on the prevalence of and interventions to reduce racial and ethnic disparities	2/2 (100 %)	Guideline or directive; identify future research needs; support program development and evaluation activities; resource allocation decisions	20 weeks	Moderate- and low-strength evidence of worse morbidity and mortality outcomes for some racial minority Veterans groups compared with white Veterans.	http://www.hsrd.research.va.gov/publications/esp/HealthDisparities.cfm
			April 2015		

### Perceptions of the content

**Fig. 2:**
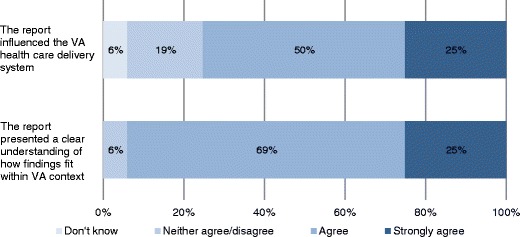
Operational partner perceptions of ESP report influence and applicability of findings

Overall, operational partners generally perceived the report content as favorable (Table 3). Eighty-one percent stated that the scope was “about right” whereas 19 % described it as “too narrow.” All operational partners “strongly agreed” or “agreed” with the findings of the report. Over half of the operational partners stated the ESP reports compared equally to other evidence sources such as Cochrane or AHRQ systematic reviews; 12.5 % preferred ESP reports for the VA focus. Regarding the restrictions on the scope and syntheses, the majority of respondents did not believe or thought they only possibly limited the usefulness of the rapid reviews. As only 27 % of respondents would have had access to other evidence sources in the absence of a rapid review, this indicates that the ESP rapid reviews have increased the VHA’s uptake of evidence for informing time-sensitive system-level decisions. We also asked operational partners if the report presented a clear understanding of how findings fit within the VA context (Fig. 2). Twenty-five percent “strongly agreed” and 69 % “agreed” with the statement. Only 6 % reported a neutral opinion on this measure.

**Table 3:** Operational partners' perceptions of report content

	Frequency
How would you describe the scope of the report?	
About right	81 %
Too narrow	19 %
To what extent do you agree or disagree with the findings of the report?	
Agree	69 %
Strongly agree	31 %
How do the ESP reports you’ve read compare with other evidence sources?	
Compare equally/similar	56 %
Prefer ESP for VA focus	12.5 %
No opinion	19 %
Other (*eg,* acknowledge benefits of different products)	12.5 %
Do characteristics of RR limit the usefulness of the report?^a^	
No	53 %
Maybe	33 %
No opinion	14 %
Yes	0 %
Without RR, how would you have addressed your research need?^a,b^	
Clinical/expert opinion	40 %
Nothing--would have had to make decision without evidence review	20 %
Used other evidence source	27 %

^a^ *N* = 15 for these measures--missing responses for one report

^b^Respondents could select multiple options for this item. Here we listed the most frequent responses.

### Resulting actions and implementation

**Fig. 3:**
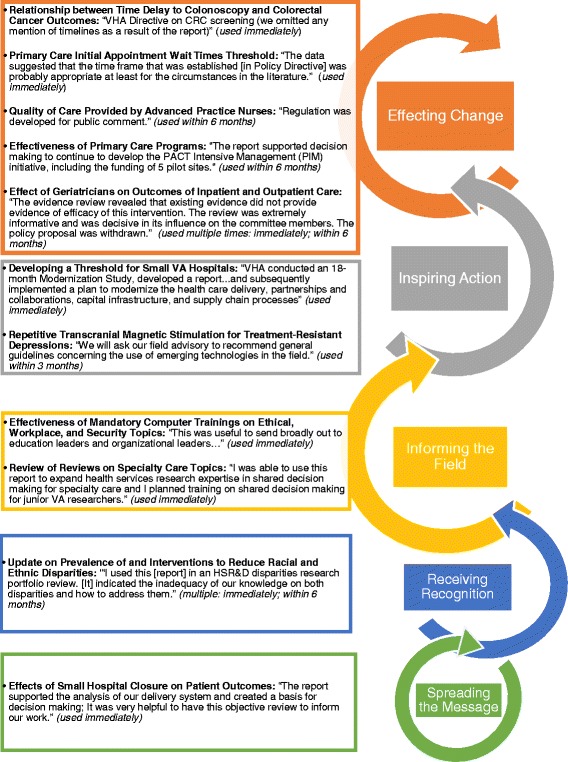
ESP reports within the IOM Degrees of Impact scale and time of use

A majority of ESP rapid reviews were used immediately and informed high-impact health system decisions. The majority of responders either strongly agreed (25 %) or agreed (50 %) that the report influenced the VA healthcare delivery system (Fig. 2). Figure 3 illustrates specific actions that resulted from the report and where these actions fall on the IOM’s Degrees of Impact scale, as well as when the report was used by operational partners. Five reports (45.4 %) yielded the highest level of impact by supporting or creating a directive on specific clinical or health systems issue. Another 18.2 % inspired action such as partnerships and plans to modernize current practices, 18.2 % informed the field in ways such as disseminating the evidence review within the field or helping plan educational trainings or meetings, 9.1 % received recognition, and 9.1 % spread a message. These qualitative perspectives in conjunction with the positive ratings on the scope, applicability, and usefulness of rapid reviews suggest that users of rapid reviews are both satisfied with the rapid product itself and use it to make decisions. Operational partners’ perceptions of rapid review content were generally homogeneous and therefore do not appear related to variability in report impact.

**Fig. 4:**
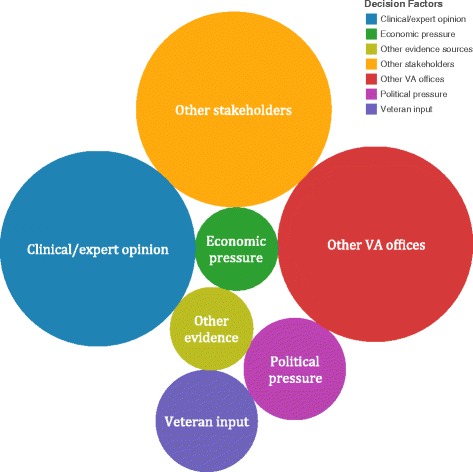
Factors influencing operational partner decision-making beyond the rapid evidence synthesis

In addition to the VA ESP rapid review, 100 % of respondents indicated that other factors influenced their decision-making process. Operational partners reported an average of 2.75 other factors that influenced the decision-making process. Figure 4 illustrates the distribution of other factors influencing decision-making which include other stakeholders (69 %), other VA offices (69 %), clinical/expert opinion (69 %), Veterans input (18.75 %), political pressure (18.75 %), economic pressure (12.5 %), and other evidence sources (12.5 %).

## Discussion

Our survey of VHA leadership has improved our understanding of how and when our VA ESP rapid reviews are being used to inform time-sensitive healthcare decision-making within the VA healthcare system. Also, these findings extend knowledge on users’ perspectives of how and when they use rapid reviews to different types of users, settings, and report topics than have been previously evaluated. Overall, operational partner feedback was positive. During its first 3 years of offering rapid reviews, the ESP program increased the uptake of evidence to inform the VHA’s time-sensitive decision-making needs, particularly on occasions where the alternative was no review of the evidence at all. The majority of ESP rapid reviews were used immediately and informed actions that ranked high on the IOM’s Degrees of Impact framework: 45.4 % effected change, 18.2 % inspired action, 18.2 % informed the field, 9.1 % received recognition, and 9.1 % spread a message. This specifically addressed the VHA’s strategic goal of rapidly translating research findings and evidence-based treatments into clinical practice [21]. Although VA rapid review topics are carefully prioritized based on a clear demonstration of urgency and presence of a mechanism for implementation, given the challenges and uncertainty of conducting rapid reviews, it is reassuring to confirm that they are being used as intended.

Our findings are consistent with previous evaluations of the impact of rapid HTAs which all found them to be valuable products [9]. Timely access to evidence and collaboration between researchers and policymakers—which are both key characteristics of rapid reviews—have frequently been reported as facilitators of implementation of evidence [4, 22, 23]. Previous research on the impact of rapid reviews has primarily focused on their use for clinical practice, policy, purchasing, and formulary decisions primarily in non-US settings [13–15]. It is useful to learn that the value of rapid reviews extends to a large US healthcare setting, such as the VA health system, for the types of process of care, access, and systems policy initiative topics addressed by the ESP rapid reviews.

It is important to note that the implementation of evidence depends not only on the content and purpose of the evidence but also on the complex environment around the topic, user, and agency [23]. Operational partners indicated that there were on average 2.75 additional factors influencing their decisions, including other stakeholders (69 %), other VA offices (69 %), clinical/expert opinion (69 %), Veterans input (18.75 %), political pressure (18.75 %), economic pressure (12.5 %), and other evidence sources (12.5 %). This suggests that our rapid reviews served as only one tool from a variety of inputs within a complex decision-making process. Learning more about the VHA decision-makers’ processes for weighing the relative contribution of rapid reviews among these different inputs, and how that may differ for standard systematic reviews, may improve our understanding of the consequences of our rapid reviews’ potential limitations.

These initial results have some limitations that we plan to address in future quality improvement efforts. First, although our operational partners’ feedback was very positive overall, this needs to be taken in context with our low response rate. However, the similarity between nonresponders and responders in their organizational roles does not clearly suggest any obvious differences in their perceptions of the reviews. It is also possible that our low response rate may be due in part to our minimal efforts to remind participants to respond to the survey. For example, although we discovered that 16 % of our nonresponders were not reachable because they had retired or were no longer with the VA, we made no further attempts to contact them. Further, we only reminded participants once via email. This was fewer than the three reminders used in the recent University of Pennsylvania Health System’s Center for Evidence-based Practice survey that had a higher response rate (72 %) [8]. We also did not employ the use of telephone reminders which have a known association of increased response rates (77 vs 53 %, *P* < .001) [24]. But the possibility of nonresponse bias remains, as other unknown differences between nonresponders and responders could exist.

Second, the retrospective nature of our preliminary data collection may raise the risk of recall bias for some of the survey items. As actions resulting from the rapid reviews are objective and a matter of record, survey items measuring impact likely have the lowest risk of recall bias. However, for survey items measuring the VHA leaderships’ perception of report content, the risk of recall bias may be greater, particularly for the older reports. We attempted to reduce this risk by providing copies of the reports along with the survey; however, it is ultimately unknown how familiar respondents were with the reports’ contents. For all future rapid reviews, we plan to address this issue by routinely surveying users only 6 months after the review’s completion. Third, although we made progress in assessing our operational partners’ acceptance of some of the potentially important trade-offs of rapid reviews (i.e., restricted scopes and syntheses), we have not yet addressed their perceptions of other specific methods to streamline the systematic review process. Empiric evidence is sparse and mixed about whether rapid reviews have less-accurate findings than systematic reviews because they often do not meet all the accepted methodological standards of standard systematic reviews [9]. For this reason, further investigation of the consequences of various methodological shortcuts continues to be among the top three key areas of interest for future rapid review research topics [11]. Fourth, our survey did not specifically assess how well we educated operational partners about and reported on the specific methodological alterations we made to gain efficiency and their potential ramifications. In the general interest of transparency and reporting guideline adherence, and because user education was a theme that emerged from AHRQ’s EPC Program interviews of potential rapid review users, this also warrants further consideration [12]. Finally, our findings should be interpreted as preliminary as our small sample size may have limited the reliability of our findings. We plan to continue surveying a larger number of users over the next several years, which will increase confidence in our findings and allow a more thorough evaluation of potential sources of variation in use and impact.

## Conclusions

Retrospective survey results preliminarily suggest that VA ESP rapid reviews have increased the VHA’s uptake of evidence for time-sensitive healthcare decision-making. The majority of ESP rapid reviews were used immediately and informed high-impact VHA decision-making. Key areas of interest for future evaluation include further assessment of users’ perceptions of specific methods we used to streamline the systematic review process and the quality of our efforts to educate about and report on such methods. Another important next step is to compare the usability and impact of VA ESP rapid and standard systematic reviews in meeting VHA leadership operational partner needs.

## Abbreviations

AHRQ, Agency for Healthcare Research and Quality; ESP CC, Evidence-based Synthesis Program Coordinating Center; ESP, Evidence-based Synthesis Program; HTA, health technology assessment; IOM, Institute of Medicine; QUERI, Quality Enhancement Research Initiative; SME, Subject-Matter Experts; VA, Veterans Affairs; VHA, Veterans Health Administration

## Additional file

Additional file 1: Survey Instrument. Copy of survey instrument sent to operational partners. Included for audience to reference if needed. (PDF 222 kb)
