# Supplementary material for: User survey finds rapid evidence reviews increased uptake of evidence by Veterans Health Administration leadership to inform fast-paced health-system decision-making
Source: Syst Rev. 2016 Aug 5;5:132. doi: 10.1186/s13643-016-0306-5 (PMC4974754; doi:10.1186/s13643-016-0306-5)
Supplement: Additional file 1: — Survey Instrument. Copy of survey instrument sent to operational partners. Included for audience to reference if needed. (PDF 222 kb) [file 13643_2016_306_MOESM1_ESM.pdf]

Supplemental Material: Survey Instrument

This survey asks about your experience with the Evidence-based Synthesis Program [ESP] and the impact of the report you received. The survey should take no longer than 15 minutes to complete. Your feedback is appreciated.

1. Select the option that best fits the need for your evidence synthesis. Check all that apply.

    a. Performance Measure
    b. VHA Guideline or Directive
    c. Clinical guidance
    d. Identify future research needs
    e. Update existing review
    f. Evaluate new technology
    g. Formulary guidance
    h. Training and curriculum development
    i. Determine implementation strategy best suited for the VHA
    j. Support program development and evaluation activities
    k. Support resource allocation decisions
    l. Other (please specify):______________________________________

2. What was your level of involvement during the review process? Check all that apply.

    a. I had periodic contact with the ESP throughout the review process to discuss report progress.
    b. I provided the ESP with input on the scope of the review.
    c. I gave the ESP feedback on the draft report.
    d. The ESP briefed me on the report's findings.
    e. I gave feedback on the draft Management eBrief (if applicable).
    f. I participated in a cyber seminar to discuss the policy implications of the report (if applicable).
    g. None

3. Please describe your experience using systematic reviews to inform health care management and policy-making.

    [Open ended]

4. How would you describe the scope of the ESP report?

    a. Too narrow
    b. About right
    c. Too broad

5. The report presented a clear understanding of how findings apply within VA context.

    a. Strongly Agree
    b. Agree
    c. Neither Agree/Disagree
    d. Disagree
    e. Strongly Disagree

6. To what extent do you agree or disagree with <u>the findings</u> of the report?

    a. Strongly Agree
    b. Agree
    c. Neither Agree/Disagree
    d. Disagree
    e. Strongly Disagree
    f. Comments:____

7. The report influenced the VA healthcare delivery system.

    a. Strongly Agree
    b. Agree
    c. Neither Agree/Disagree
    d. Disagree
    e. Strongly Disagree
    f. Don't know

8. How do the ESP reports you've read compare with other evidence sources (e.g., Cochrane, AHRQ)?

    [Open ended]

9. How soon did you use the report after receiving it?

    a. Immediately (less than one month)
    b. Within 3 months
    c. Within 6 months
    d. Within 1 year
    e. Over a year

    f. Did not use report
        i. **If respondents select "did not use report," survey skips to*
            1. Please provide more detail on why you did <u>not</u> use report.

                [Open-ended]

2. How likely are you to use ESP results to develop new VHA policies or clinical practices?
    a. Very likely
    b. Likely
    c. Somewhat likely
    d. Not likely
    e. Don't know

10. *Only asked of those that used report:* What actions resulted from the report to address the needs identified in Question 1? Please be as specific as possible. For example, if the report supported your decision-making, what was your final decision?

    (Responses included: performance measure, VHA guideline/directive, clinical guidance, identify future research needs, update existing review, evaluate new technology, formulary guidance, training/curriculum development, determine implementation strategy, support program development/evaluation activities, support resource allocation decision, other)

    [Open ended]

11. What other factors influenced the decision-making process? Check all that apply.

    a. None
    b. Veteran input
    c. Other stakeholders
    d. Other VA offices
    e. Political pressure
    f. Economic pressure
    g. Clinical/expert opinion
    h. Other evidence sources (e.g. Cochrane, AHRQ)
    i. Did not use report
    j. Additional information:____

12. How useful was the cyber seminar for disseminating findings to the field?

    a. Very useful
    b. Fairly useful
    c. Slightly useful
    d. Not useful at all
    e. No opinion
    f. Not applicable
    g. Comments:____

13. How useful was the Management eBrief for disseminating findings to the field?

    a. Very useful

b. Fairly useful
c. Slightly useful
d. Not useful at all
e. No opinion
f. Not applicable
g. Comments:____

14. In terms of dissemination, besides the Management eBrief and cyber seminar, did you request additional ESP presentations of the report's findings to other audiences?

a. No
b. Yes
    i. If yes, please specify:_______________________

15. I am likely to request reports from ESP in the future.

a. Strongly Agree
b. Agree
c. Neither Agree/Disagree
d. Disagree
e. Strongly Disagree
f. Not applicable

16. Are you aware of any new studies that have impacted the strength or direction of the evidence that might affect the report's conclusions <u>to the extent that it should be updated</u>?

a. Yes
b. No
c. Additional comments:_______________________________

****Rapid Review Users Only****

17. Rapid Reviews generally have a narrower scope and less synthesis as compared to a full systematic review. Do you believe these characteristics limit <u>the usefulness</u> of Rapid Review?

a. Yes
b. No
c. Maybe
d. No opinion

18. If the ESP had been unable to do a Rapid Review on this topic, how would you have addressed your research need?

a. Nothing—would have had to make a decision without a review of the evidence
b. Used other evidence source
c. Clinical/expert opinion
d. Other:_________________________________
e. Not applicable

Comments and Suggestions

Please leave any additional comments that you feel are important to the growth of ESP and the reports we produce.
